# Switching of spins and entanglement in surface-supported antiferromagnetic chains

**DOI:** 10.1038/s41598-017-02972-x

**Published:** 2017-06-05

**Authors:** Ilia N. Sivkov, Dmitry I. Bazhanov, Valeri S. Stepanyuk

**Affiliations:** 1 Max Planck Institute of Microstructure Physics, Halle, 06120 Germany; 2 ETH Zürich/CSCS Lugano, Lugano, Switzerland; 3 Faculty of Physics, Moscow State University, GSP-1, 119991 Moscow, Russia; 4 Dorodnicyn Computing Centre, FRC CSC RAS, Vavilov st. 44, b. 2, 119333 Moscow, Russia

## Abstract

Previous experimental studies discovered universal growth of chains and nanowires of various chemical elements on a corrugated molecular network of Cu_3_N on the Cu(110). Herein, performing combined *ab initio* and quantum Hamiltonian studies we demonstrate that such chains can be used for a fast spin switching and entanglement generation by locally applied magnetic pulses. As an example, we show that in antiferromagnetic Co chains a strong entanglement between ends of chains occurs during spin switching. A novel parity effect in spin dynamics is reported. Even-numbered chains are found to exhibit significantly faster spin switching than odd-numbered counterparts. Moreover, at certain parameters of the system the dimerization effect in the spin dynamics of the chains was found. Our studies give a clear evidence that tailoring spin dynamics and entanglement can be achieved by magnetic fields and by tuning exchange interactions in supported chains.

## Introduction

The emerging field of quantum engineering has the potential to create new quantum technologies. Quantum entanglement between spins is considered as a key resource for such future applications^1^. Spin chains are promising candidates for the generation of entanglement and qubit teleportation^2–8^, The transfer of a classical information along a quantum spin chain has been found to strongly depend on the quantum magnetic phases in the chain which can be tuned by the transverse magnetic field^9^. Antiferromagnetic spin chains can exhibit entanglement over long distances^10, 11^, The entanglement in antiferromagnetic chains can be increased by increasing the temperature or the external field^12^. Several experiments have shown that entanglement in atomic-scale nanostructures on surfaces can survive at low temperatures even in the presence of decoherence caused by the environment^13–15^, To reduce the decoherence insulating substrates can be used^16^. For example, thin insulating layers, specifically CuN_2_ on Cu(001) surface, provide significant decoupling between spins in surface-supported nanostructures and the bulk electrons in the metal below, resulting in relatively long-lived spin states^17, 14^, Yan et al^17^. have observed very long lifetimes in the case of antiferromagnetic Fe_3_ chains on CuN_2_. Gauyacq and Lorrente^18^ have shown that decay from one state to another in such system strongly depends on the degree of entanglement of the local spins in the chain. Recent experiments on engineered small magnetic units on CuN_2_ have demonstrated the possibility to study and control their spin states which is a very significant step towards atomic-scale memories^19–22, 15^, Moreover, it has been^19^ shown that data can be stored in antiferromagnetic atomic-scale structures on CuN_2_ allowing to achieve data storage densities that are about 100 times higher than those in modern hard drives^22^. In very recent remarkable experiments of Choi et al^13^. the possibility to tune entanglement in chains of magnetic adatoms on a CuN_2_/Cu(001) surface was demonstrated. Spin chains with defined entanglement can be produced by changing the composition and coupling within the chains.

**Figure 1:**
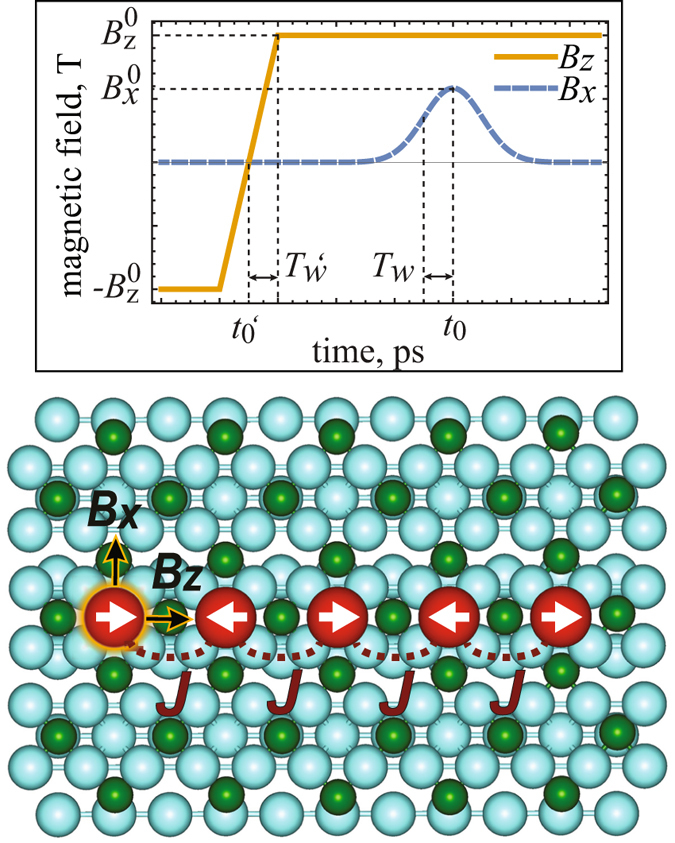
Upper inset: Time-dependent magnetic pulse *B*_*x*_ and field *B*_*z*_ applied to the edge atom in the chain. The step-like reversible field *B*_*z*_ is determined by amplitude ${B}_{z}^{0}$ and ascribed by a center of filed ${t}_{0}^{^{\prime} }$ (set at 30 *ps*) with a half-width ${T}_{w}^{^{\prime} }$ (set at 10 *ps*), whereas Gaussian-like pulse *B*_*x*_(*t*) is determined by amplitude *B*_*x*_^0^ (set at 0.5 *T*) and ascribed by a shift of the pulse *t*_0_ (set at 100 ps) with a half-width *T*_*w*_ (set at 10 *ps*). Lower inset: Schematic view of a finite cobalt chain (Co_5_) placed on a self-corrugated Cu_3_N/Cu(110) surface. The red (large dark) spheres indicate the Co atoms, the green (small dark) spheres indicate the N atoms, and the turquoise (light) ones indicate the Cu atoms, respectively. The exchange parameter *J* indicates spin coupling between cobalt atoms in neighboring sites.

In all above mentioned experiments, spin chains on CuN_2_ were created in control way by atomic manipulation with scanning tunnelling microscopy (STM). Besides, there is another copper nitride insulating layer studied by STM - a self-corrugated Cu_3_N nitride phase on a Cu(110) surface, which forms a covalently polar bonded molecular network similar to the copper nitride (CuN_2_) on a Cu(001) surface^23^. This Cu_3_N network served as a perfect template for universal growth of atomic nanowires with uniform width and height among various transition metal elements. Moreover, the structure information, obtained by STM at the initial stage of epitaxial growth, revealed the universal growth of small linear chains distributed randomly at the bumping area within a trough of a self-corrugated Cu_3_N network (see Fig. 1 and^2^ for more details). Our recent *ab initio* and quantum spin Hamiltonian studies have predicted that these antiferromagnetically coupled spin chains on Cu_3_N can exhibit the quantum entanglement up to rather high temperature (20–100 K)^2^. Therefore, we believe that such a built spin chains on Cu_3_N are a new playground for exploring quantum magnetic phenomena at the atomic scale.

Here, combining *ab initio* and quantum spin Hamiltonian studies, we demonstrate that antiferromagnetically coupled Co_*n*_ chains of various lengths (*n* - number of cobalt atoms) on a Cu_3_N/Cu(110) can be used for a fast spin switching and generation of entanglement by the external magnetic pulses. In particular, the emergence of strong entanglement during spin switching is revealed. It is shown that propagation of this entanglement through cobalt chains can be tailored effectively by magnetic pulses and by tuning the exchange interactions between atomic spins. We have also found that for small exchange interactions the dimerization effect in the spin dynamics could be observed. Our results indicate that engineering of spin switching and entanglement in atom-by-atom fashion can be achieved by exploiting the parity effect of an even- and odd-numbered Co_*n*_ chains, which exhibit significantly different quantum states. Our results offer a promising way to design spin-information transfer and entanglement of spin qubits based on atomic spin chains on insulating supports.

## Parity effect and spin dynamics

To study the quantum spin dynamics of the finite Co_*n*_ chains on a Cu_3_N/Cu(110) surface (to be referenced further as Cu_3_N in this paper), we performed *ab initio* calculations of the chains up to seven cobalt atoms (*n* = 2.7) placed in the hollow sites of the bumping area of self-corrugated copper nitride surface layer aligned along[110] (or *Z* axis in our case) direction and repeated periodically by means of a supercell approach (see the Methods and Fig. 1 for details). The arrangement of Co_*n*_ chains on a Cu_3_N surface leads to the separation of their nearest neighbors within a chain with a distance of about 5 Å, while the next-nearest neighbors are far apart with a distance more than 10 Å. Such large separation results in a substantial decay of the exchange interaction for the next-nearest neighbors, which becomes almost negligible. We therefore constrained ourselves here for treatment of only the nearest neighbors exchange interactions in Co chains. In previous work^2^ we showed, that intrachain exchange interaction is mediated by the nitrogen atoms interposing from the underlying Cu_3_N network and thereby exhibits a superexchange-like character. It is giving rise to a strong antiferromagnetic coupling of atomic spins with antiparallel alignment along the chain. The non-collinear calculations, including spin-orbital correction, allowed us to estimate quantitatively the strength of this exchange coupling (*J* = −0.352 *meV*) together with uniaxial (*D* = −0.352 *meV*) and transverse (*E* = 0.069 *meV*) anisotropies. Having received these values, we can make a setup to spin dynamics simulations of cobalt chains on a Cu_3_N surface in the framework of our model spin Hamiltonian.

However, in order to trigger the spin relaxation process, we have perturbed a spin state of an edge atom in the cobalt chain by applying an effective external magnetic field, which, for instance, occurs in close proximity of spin-polarized STM tip^17^ or neighboring small cluster^24^. Such a magnetic field can probe locally a spin on the atomic scale and can cause a time evolution of a spin state propagating across the cobalt chain^25^. To achieve that we have applied to the edge atom the time-dependent external magnetic field *B*_*z*_(*t*) ascribed by step-like function (see inset in Fig. 1) with a reversible magnetization from ${B}_{z}^{0}$ to − ${B}_{z}^{0}$ along *Z* direction (along easy magnetization direction of a chain). Besides, due to the high symmetry of a spin structure along *Z* direction, we have applied also a short Gaussian magnetic pulse ${B}_{x}(t)={B}_{x}^{0}\exp [-\frac{1}{2}{(\frac{t-{t}_{0}}{{T}_{w}})}^{2}]$ along *X* direction in order to break such symmetry and to accelerate a time evolution of the spins for the considered chains (see inset in Fig. 1). The amplitude of *B*_*x*_ pulse and its width used in the present work are available with a current technology^26, 27^, Such pulse can correspond to the magnetic component of THz radiation. Strong (a few T) and very short (2–4 ps) magnetic pulses can also be generated using relativistic electron bunches^28, 29^, We reverse the direction of *B*_*z*_ (Fig. 1) over timescale of picoseconds. In future experiments it could be possible using ultrafast optical manipulation of magnetic states^30^. To sum up, we can write the total Hamiltonian of the system as:

1 $$\hat{H}=-2J\sum _{i=1}^{N-1}{\hat{\overrightarrow{S}}}_{i}{\hat{\overrightarrow{S}}}_{i+1}+\sum _{i=1}^{N}[D{\hat{S}}_{i,z}^{2}+E({\hat{S}}_{i,x}^{2}-{\hat{S}}_{i,y}^{2})]-g{\mu }_{B}[{B}_{z}(t){\hat{S}}_{1,z}+{B}_{x}(t){\hat{S}}_{1,x}],$$

here ${\hat{\overrightarrow{S}}}_{i}$ is the spin operator on the *i* th site in the chain (has spin magnitude *S* = 3/2 for Co), whereas *g* is the Landé factor (with free-electron value *g* = 2.00) and *μ*_*B*_ is the Bohr magneton. The choice of the Co spin quantum number *S* = 3/2 is justified by the fact that *ab initio* calculations show a total spin on cobalt atom and ligand atoms to be close to 3/2, which is in agreement with an experimental Kondo-effect observed on cobalt atom^31^.

One should note that the ground state of the even-numbered antiferromagnetic chains is the singlet with S = 0, characterized by a wave function in which all spins populate opposing spin states equally^32–34, 22^. Neel states are broken-symmetry solution of the Heisenberg Hamiltonian (HH) (Eq. (1)), i.e. the Neel state is not the ground state of the antiferromagnetic HH^33^. A small perturbation acting on one of the spin of the chain is enough to split the ground states into two Neel-like states. This perturbation can be of various origins (small inhomogeneities of a surface, external magnetic field induced by the spin-polarized tip etc.). The emergence of the Neel state from the ground AF state caused by measurements has been revealed in theoretical studies^33, 34^. The presence of a decoherence makes also the two Neel states the equilibrium states of the system^32, 22, 35^. In the absence of decoherence(free chains) (or at extremely low temperatures if decoherence becomes negligible) the Rabi oscillations between two Neel states occur^32^. In our case the perturbation of the system is governed by external magnetic field which is applied only to the first spin. This field breaks the symmetry of the system and leads to the existence of Neel-like states.

**Figure 2:**
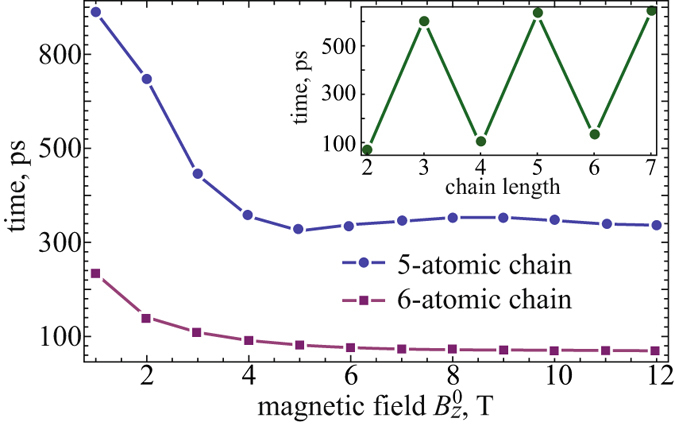
The evolution of relaxation time for 5-and 6-atomic chains locally perturbed at the edge by external magnetic field *B*_*z*_. Inset: Relaxation time for the set of Co_*n*_ spin chains with *n* = 2.7 under magnetic field ${B}_{z}^{0}$ = 2 *T*.

Now we turn to the discussion of the time evolution of magnetization in cobalt chains. First, in Fig. 2 we present the relaxation time of atomic spins caused by a magnetic field with a reversible amplitude ${B}_{z}^{0}$ = 2 *T* and demonstrate its evolution as a function of this amplitude for 5- and 6-atomic chains, as a particular example. The relaxation time has been determined as a time when a spin structure of the system reaches a nearly equilibrium state with a deviation of the total energy from the initial one less than 1%. As clearly seen in inset of Fig. 2, the relaxation time significantly differs for the chains with even and odd numbers of spins, which decreases for higher fields and saturates to less than a half of the value for the low fields. In Fig. 2 we plotted, as an illustration, the corresponding curves with asymptotical limits around ~350 *ps* and ~100 *ps* obtained for 5- and 6-atomic chains, respectively. To gain insight into such dynamical behavior of the even- and odd-numbered chains (so-called parity effect) we explored the peculiarities of their ground state. As it was mentioned above, an even-numbered antiferromagnetic chain has zero total spin and a singlet ground state, while an odd-numbered antiferromagnetic chain in general has non-zero total spin and, therefore, degenerate ground state. This degeneracy can be removed by applying external magnetic field leading to the spin arrangement in the system^22^. Thereby, when the magnetic field with a reversible magnetization is applied to an odd-numbered chain, the chain undergoes the transition between spin states triggered by field switching from + *Z* to − *Z* direction. Therefore the matrix element of the perturbed Hamiltonian between these states $\langle {\Psi }_{B\uparrow }|{\hat{H}}_{B\downarrow }|{\Psi }_{B\downarrow }\rangle  \sim \langle {\Psi }_{B\uparrow }|{\Psi }_{B\downarrow }\rangle =0$, and transition can occur only due to the relaxation term (see Eq. (2) in the Methods). If the damping constant *λ* is small (in our case *λ* = 0.05), then the relaxation time is quite large (about several hundreds picoseconds). For the case of an even-numbered chain, the same external magnetic field can only slightly modifies a singlet ground state, which is fully entangled and with the matrix element $\langle {\Psi }_{B\uparrow }|{\hat{H}}_{B\downarrow }|{\Psi }_{B\downarrow }\rangle \sim \langle {\Psi }_{B\uparrow }|{\Psi }_{B\downarrow }\rangle \ne 0$. To confirm these statements we perform at the end of this paper (see the Methods) an analytical description of parity effect for model 2- and 3-atomic chains with spin *S* = 1/2, as for the simplest representatives of even- and odd-numbered chains.

Finally, we comment on the importance of *B*_*x*_. As our calculation shows, *B*_*x*_-time *t*_0_ (see Fig. 1) with respect to the *B*_*z*_-time ${t}_{0}^{^{\prime} }$ influences on the behavior of the odd-numbered chains: the closer *t*_0_ to ${t}_{0}^{^{\prime} }$, the earlier the chain flips (see Supplementary information, Fig. S1). At the same time *B*_*x*_ gives no effects for the even-numbered chains since the switch occurs almost at the time of the switch of *B*_*z*_ due to a fast tunnelling between |Ψ_*B*↑_〉 and |Ψ_*B*↓_〉 states (as it is mentioned above).

### Entanglement and magnetic field dependence

**Figure 3:**
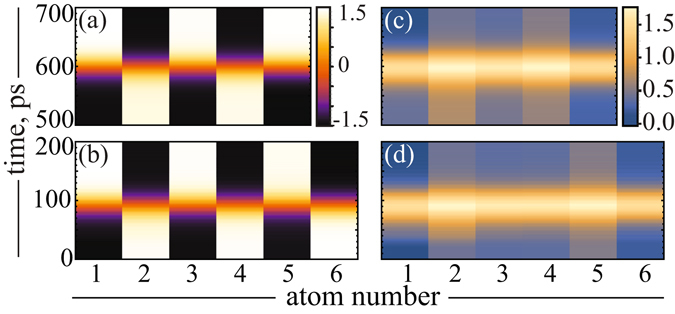
Time evolution of <*S*_*z*_> spin components [(**a**) and (**b**)] and the calculated single-particle Von Neumann entropies [(**c**) and (**d**)] for the 5- and (**b**) 6-atomic chains, respectively.

**Figure 4:**
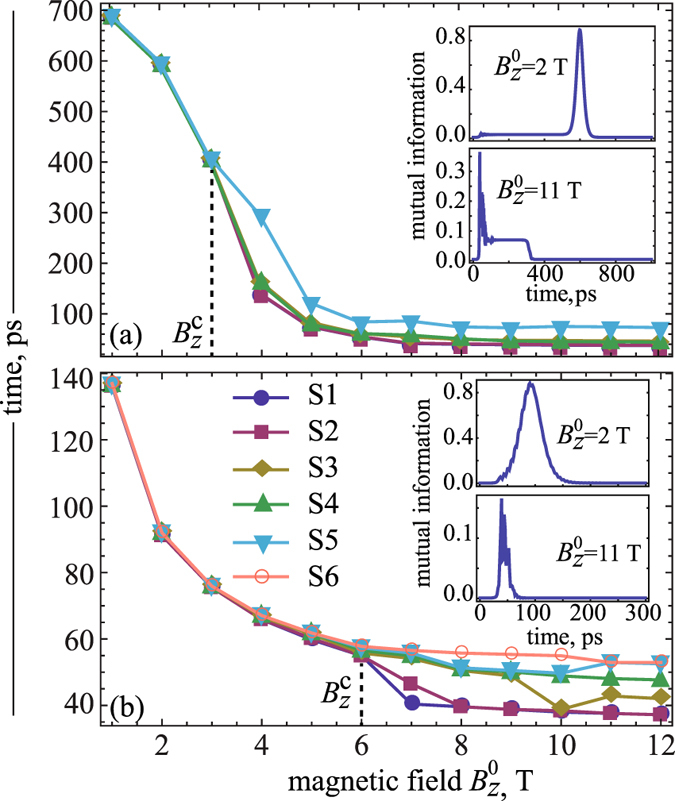
Dependence of a spin reversal time [(**a**) and (**b**)] for 5- and 6-atomic chains on the external magnetic field amplitude ${B}_{z}^{0}$, respectively. Insets: Time evolution of two-particle mutual information between edge spins of the corresponding chains under the fields *B*_*z*_ with ${B}_{z}^{0}$ = 2 *T* and ${B}_{z}^{0}$ = 11 *T* (for the 5-atomic chains the effects of *t*_0_ of *B*_*x*_ and amplitude *B*_*x*_^0^ are found, see Supplementary information, Figs S2 and S3).

Recent theoretical^2^ and experimental^13^ studies assigned the existence of the quantum entanglement of the spins among antiferromagnetic 3*d* chains on a copper nitride surfaces for realistic conditions. We demonstrate here how the parity effect can affect this entanglement for the considered cobalt chains. First of all, in Fig. 3 we present the time evolution of <*S*_*z*_> spin components and the single-particle Von Neumann entropies (see Eq. (4) in the Methods) obtained for each atomic spins within 5- and 6-atomic cobalt chains, and for the field ${B}_{z}^{0}\mathrm{=2}\,T$. It is well seen, that a single-particle entropy of each atomic spin is rather small before and after the time of the spin-flip (Fig. 3(c,d)). Furthermore, during the spin-flip transition all <*S*_*z*_> spin components are passing through zero. We established in this case that the expectation value of the total spin $< {\hat{S}}^{2} >$ ($\hat{\overrightarrow{S}}={\sum }_{i=1}^{n}{\hat{\overrightarrow{S}}}_{i}$) is negligible and, thereby, we concluded that both chains should exhibit a high entangled spin states^6^. This conclusion is corroborated by enhancement of entropies observed for both chains during the spin-flip transition (Fig. 3(c,d)), which is related directly to quantum entanglement of spins. This quantum behavior of spins becomes quite interesting, if we increase the magnitude of the magnetic field ${B}_{z}^{0}$. In Fig. 4 we present results of our calculations, which demonstrate the dependence of a spin-flip time (when <*S*_*z*_> changes its sign) on ${B}_{z}^{0}$ obtained for each spin within 5- and 6-atomic chains as a function of the magnetic field magnitude. One can see, that below some “critical” magnetic field ${B}_{z}^{c}$ (3 *T* and 6 *T* for 5- and 6-atomic chains, respectively) the spins flip practically simultaneously (see also Fig. 3), while above this field the spins have different switching times. In another words, for magnetic fields less then the critical ${B}_{z}^{c}$ one can speak about fast spin switching. Analysis of spin dynamics among cobalt chains revealed a strong oscillations of <*S*_*z*_> spin components. These oscillations exhibit the asynchronous time evolution observed for the magnetic fields ${B}_{z}^{0}$ > ${B}_{z}^{c}$, that signifies obviously the impact of the field on the dynamical correlations within spin chains.

To get a quantitative confirmation of this conclusion, we performed the calculations of the two-particle mutual information (related to two-particle Von Neumann entropies, see Eq. (6) in the Methods) of the chain edge atoms, as a particular example, for the small and large fields. This information allows one to evaluate a strength of a quantum correlation between the spins at the edges during propagation of a spin-flip signal along the chain. The calculations of the mutual information for the edge atoms in both considered chains clearly show that for large magnetic fields (${B}_{z}^{0}$ > ${B}_{z}^{c}$ = 11 *T*) the correlation is much less than for small fields (${B}_{z}^{0}$ < ${B}_{z}^{c}$ = 2 *T*) (see insets in Fig. 4) and zero before and after the switching. It means that the large magnetic field weakens spin correlation between edge atoms and, consequently, decreases entanglement of their spins. It is important to note that in our case in the ground state for the even numbered chains total magnetization is zero, but <*S*_*z*_> component on each spin is not zero. As it is seen in Fig. 3(b) this is described by the fact that before and after the switching the chain forms Neel-like state with a small onsite Von-Neumann entropy. However during the switching process a strong enhancement of the entanglement and edge-to-edge mutual information is observed for moderate magnetic fields (see Figs 4 and S4). In this case the Neel-like state is lost and all spins are in highly-entangled states.

**Figure 5:**
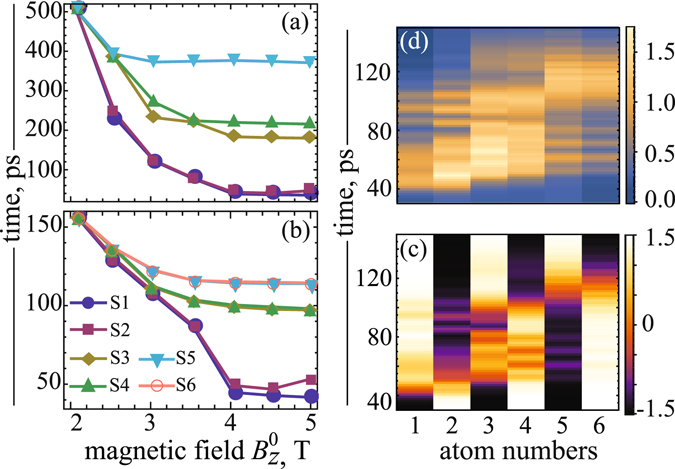
Dependence of a spin reversal time [(**a**) and (**b**)] for 5- and 6-atomic chains, respectively, with exchange parameter *J* = − 0.1 *meV* on the external magnetic field amplitude ${B}_{z}^{0}$. Diagrams: (**c**) spin dynamics of <*S*_*z*_> spin components and (**d**) a single-particle Von Neumann entropies between neighboring spins for 6-atomic chain, respectively.

Finally, we examine how the coupling strength between spins can effect the quantum entanglement in chains. It was shown by recent STM study^13^, that the coupling strength and its ratio with the magnetic anisotropy energy (*J*/|*D*|), can be the dominant features responsible for existence of spin entanglement in the antiferromagnetic chains on a copper nitride surface. Therefore, we carried out the series of spin dynamics simulations by decreasing the exchange parameter (*J*) value with respect to the values of anisotropy constants (*D* and *E*). Here, we followed the approach provided by recent STM studies of Fe and Co spin chains on a Cu_2_ N surface, where the authors were able to tune the strength of the exchange interaction within a chain by adjusting the relative position and orientation of the individual atoms on the underlying surface layer^36, 16, 37, 20^, In Fig. 5 we present the results obtained for spin dynamics of 5- and 6-atomic cobalt chains with parameter *J* = −0.1 *meV*. We treat here only this exchange interaction, because it corresponds to the available experimental data established for interacting spins with a large separations^16^. It can be used without limiting the generality of obtained results and conclusions, since, having changed the exchange parameter within the same order of magnitude, we didn’t observe any qualitative differences in spin dynamics among considered chains. Figure 5(a,b) shows the time evolution of a spin reversal within cobalt chains for a set of applied magnetic fields. Our key finding here is that during spin dynamics the chains are dimerized through formation of spin pairs (such as S1-S2, S3-S4, and S5-S6). It is well seen, that spin switching of atoms in chain occurs at substantially different timescales: it occurs with a significant time delay between neighboring pairs of spins, but almost simultaneously within each spin pair. Note, that the critical field ${B}_{z}^{c}$ is strongly reduced by comparing the results presented in Fig. 4. The decreasing of the exchange coupling *J* between spins weakens their entanglement^13^. The quantum entanglement of AFM spin chains is decreased by the increasing of the external magnetic field *B* at zero temperature^12^. Therefore, the notable reduction of a critical field ${B}_{z}^{c}$ for smaller *J* is mainly caused by the decreasing of entanglement in the considered chains.

**Figure 6:**
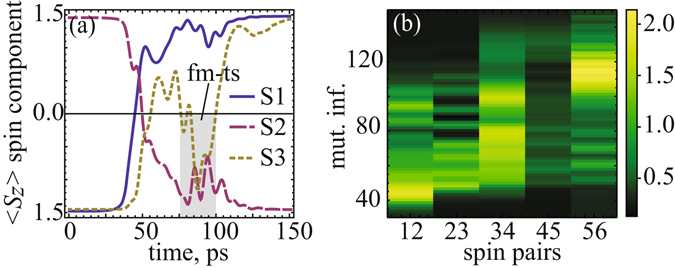
(**a**) Time evolution of <*S*_*z*_> spin components for *S*1, *S*2 and *S*3 spins of 6-atomic chain. Inset: the transient ferromagnetic-like state (fm-ts) for a time ~25 *ps* is observed between *S*2 and *S*3 spins within the time range from ~75 *ps* to ~100 *ps*. Diagram (**b**): two-particle mutual information between neighboring spins in considered chain.

To gain insight into the origin of such a strongly non-equilibrium spin dynamics, we examine the time evolution of the <*S*_*z*_> spin components (Fig. 5(c)) and the single-particle Von Neumann entropies (Fig. 5(d)) for 6-atomic chain under external magnetic field *B* = 4 *T*. Here, one can see that the spin components and the entropy are evolving by pairs. Moreover, a careful look at Fig. 5(c) reveals an unexpected scenario of spin switching between these pairs. We found that, owing to the substantially different timescales, the spin-flip transition of neighboring spins between spin pairs occurs by a way of a transient ferromagnetic-like state. This transient state is emerging through a temporary parallel alignment of spins, in spite of their antiferromagnetic coupling in ground state (Fig. 6(a)). Remarkably, the time required for the disappearance of this non-equilibrium ferromagnetic state is rather large for considered cobalt chains. In particular, using the spin dynamics simulations with adjusted damping constant *λ* (see Eq. (2) in the Methods), we found this time to be ~25 *ps* and ~20 *ps* for *S*2-*S*3 and *S*4-*S*5 interacted spins, respectively. Note that a similar transient regime of the spin dynamics was observed experimentally during ultrafast reversal of antiferromagnetically coupled spins within two magnetic sublattices in Gd-Fe-Co system^38^. However, the lifetime of the transient state in this case is significantly smaller than in our chains due to larger exchange interactions in bulk systems. This observation supports our own findings and indicates an existence of a strong spin correlations inside the spin system. To prove this statement we calculated two-particle mutual informations, which are responsible for quantum entanglement between two spins within chain, respectively. As shown in Fig. 6(b), the calculated two-particle mutual information exhibits a large entanglement between paired spins (*S*1 − *S*2, *S*3 − *S*4, *S*5 − *S*6), but drops it gradually in between these spin pairs, when transient ferromagnetic-like state mediates spin reversal of neighboring spins (*S*2 − *S*3, *S*4 − *S*5) within chain. It is noteworthy that such a remarkable dynamical propagation of entanglement through spin pairs was already predicted theoretically for model antiferromagnetic spin chains^6^. Thus, it is reasonable to conclude that dimerization of spins reported here is an intrinsic property of the antiferromagnetically coupled spin chains. Besides, our simulations predict that mutual information for paired and unpaired spins increases rapidly during a propagation of entanglement through the spin chain. This result seems to be very attractive, since it could give a credit for supported antiferromagnetic spin chains, as a prototype *N*-spin system, for the relevant technological implementations, such as quantum computation or teleportation^39^. Key challenges related to this process are the thermal instability and quantum decoherence governing the entanglement propagation in the *N*-spin system at large scale^40, 41^, However, if entanglement propagates sufficiently fast, then decoherence doesn’t have enough time to act^42^. Furthermore, entanglement in antiferromagtic spin chains was found to be less sensitive to the temperature than in ferromagnetic ones and thermally more stable, if it is generated for spins at large separations^6, 40^, As it was shown, entanglement in this case exhibits an exponential decay with decoherence, which is much slower than in ferromagnetically coupled spins.

To conclude, our findings unveil a new insight into spin dynamics of cobalt chains antiferromagnetically coupled on insulating layer of a Cu_3_N/Cu(110) surface. It is shown that even- and odd-numbered chains exhibit spin reversal at substantially different timescales (parity effect), owing to the significantly different spin ground states. Even-numbered chains reveal much faster spin switching, than odd-numbered counterparts under the external magnetic field. Besides, the emergence of strong spin correlations and entanglement within chains is established during spin switching. Considering much weaker interaction of the spins within chains, an unexpected scenario of spin switching is observed. In this case the spin chains are dimerized through the formation of spin pairs and spin switching between them is mediated by a transient ferromagnetic-like state, in spite of antiferromagnetic spin coupling in the ground state. We believe that tapping into this quantum behavior of spin chains is very important for the understanding of both the fundamental physics and the atomic-scale limits for quantum information processing.

## Methods

### Details of *ab initio* calculations of Co_*n*_ chains on a Cu_3_N/Cu(110) surface

To examine the structural and magnetic properties of the considered cobalt chains we carried out an *ab initio* molecular dynamics calculations based on density functional theory (DFT) as it is implemented in VASP code^43^. The VASP code was used to solve the Kohn-Sham equations with periodic boundary conditions and a plane-wave basis set. An all-electron projector augmented wave (PAW) method was employed within this code for calculations of total energy and forces^44^. The electron exchange and correlation effects were taken into account using generalized gradient approximation (GGA)^45^. We used a maximal kinetic energy cutoff of 400 eV which converged iteratively the total energy of the considered systems to within 1 meV/atom. The integration over the Brillouin zone (BZ) was performed on a well converged *k*-point mesh 6*x*6*x*1, using the tetrahedron method with Blöchl corrections^46^. More technical details of the calculations one can find in our previous work^2^.

### Details of the spin dynamics simulations

To carry out spin dynamics simulations of the magnetic Co_*n*_ chains on a Cu_3_N surface, we used the Heisenberg-Dirac-Van Vleck quantum spin Hamiltonian (Eq. (1)) within the irreducible tensor operator technique^47, 48^, Also, the calculations were repeated using the direct product states basis. The single-site values of an effective exchange coupling (*J*) and magnetic anisotropy constants (*D* and *E*) were received directly from our *ab initio* calculations of total energies within the DFT approach.

The time evolution of the magnetization in supported magnetic cobalt chains was studied via the method proposed in ref. 49 where the time-dependent Schrödinger equation is solved together with a damping term as an analog to the Landau-Lifshitz equation^50^ for classical magnetic dynamics. All the calculations were performed using home-made code and repeated in the Mathematica package. According to the ref. 49 one can write the time-dependent Schrödinger equation with a relaxation term as

2 $$i\hslash \frac{d}{dt}|\psi (t)\rangle =(\hat{H}-i\lambda (\hat{H}-{\langle H\rangle }_{t}))|\psi (t)\rangle ,$$

where 〈*H*〉_*t*_ is the energy of the system at time *t*. It was shown^49^, that for the Heisenberg systems this equation can be reduced to the Landau-Lifshitz equation. In our case we will use an analogy of the Landau-Lifshitz-Gilbert equation replacing *t* → *t*/(1 + *λ* ^2^)

3 $$i\hslash \mathrm{(1}+{\lambda }^{2})\frac{d}{dt}|\psi (t)\rangle =(\hat{H}-i\lambda (\hat{H}-{\langle H\rangle }_{t}))|\psi (t)\rangle ,$$

Numerically this equation was solved by Runge-Kutta method.

As a quantity of the entanglement degree of a single particle in a many-body system one can use the Von Neumann entropy, which is calculated as

4 $$S({\hat{\rho }}_{1})=-Tr({\hat{\rho }}_{1}lo{g}_{2}{\hat{\rho }}_{1})=-\sum _{{m}_{1}}{\lambda }_{{m}_{1}}{\mathrm{log}}_{2}{\lambda }_{{m}_{1}},$$

here ${\hat{\rho }}_{1}$ is a reduced density matrix of 1st particle and *λ*_*m*1_ is its *m*_1_ eigenvalue. The reduced density matrix is calculated from the total density matrix of a pure state taking a trace over the indices of the other particles

5 $${\hat{\rho }}_{1}=T{r}_{\mathrm{2,3,}\mathrm{...}}(|\psi (t)\rangle \langle \psi (t)|)=T{r}_{\mathrm{2,3,}\mathrm{...}}(\hat{\rho }\mathrm{).}$$

Additionally, we calculate the mutual information between spins in the chain as

6 $${I}_{i,j}=S({\rho }_{i})+S({\rho }_{j})-S({\rho }_{i,j})$$

which shows correlation between particles.

### Parity effect for model spin chains with S = 1/2: analytical description

Here, we describe analytically the parity effect dealing with 2- and 3-atomic chains with spin *S* = 1/2 for simplicity. For 2-atomic chain our model spin Hamiltonian (Eq. (2)) can be written schematically as

7 $$\hat{H}=-2J{\hat{\overrightarrow{S}}}_{1}{\hat{\overrightarrow{S}}}_{2}-B{\hat{S}}_{\mathrm{1,}z},$$

with *J* < 0 for antiferromagnetic exchange coupling and under external magnetic field *B*. When the field is absent (*B* = 0), a singlet ground state of the system is ascribed by

8 $$|{\Psi }_{0}\rangle =1/\sqrt{2}(|\uparrow \downarrow \rangle -|\downarrow \uparrow \rangle ),$$

while in the presence of the field (*B* ≠ 0) the ground state will be ascribed using the first order of the perturbation theory (|*B*/*J*| ≪ 1) by

9 $$|{\Psi }_{B}\rangle =\frac{1}{\sqrt{2}}[(1+\frac{B}{4J})|\uparrow \downarrow \rangle -(1-\frac{B}{4J})|\downarrow \uparrow \rangle ].$$

From here one can get the product 〈Ψ_*B*↑_|Ψ_*B*↓_〉 = 1 − (*B*/4*J*)^2^.

In the same manner, for the 3-atomic chain we can write a model spin Hamiltonian as

10 $$\hat{H}=-2J({\hat{\overrightarrow{S}}}_{1}{\hat{\overrightarrow{S}}}_{2}+{\hat{\overrightarrow{S}}}_{2}{\hat{\overrightarrow{S}}}_{3})-B{\hat{S}}_{\mathrm{1,}z}.$$

Then it can be easily shown in the framework of the perturbation theory that the ground states for *B*_*z*_ > 0 and *B*_*z*_ < 0 look like

11 $$|{\Psi }_{B\uparrow }\rangle ={\alpha }_{1}|\uparrow \uparrow \downarrow \rangle +{\alpha }_{2}|\downarrow \uparrow \uparrow \rangle +{\alpha }_{3}|\uparrow \downarrow \uparrow \rangle ,$$

12 $$|{\Psi }_{B\downarrow }\rangle ={\tilde{\alpha }}_{1}|\downarrow \downarrow \uparrow \rangle +{\tilde{\alpha }}_{2}|\uparrow \downarrow \downarrow \rangle +{\tilde{\alpha }}_{3}|\downarrow \uparrow \downarrow \rangle ,$$

where *α*_*i*_ and ${\tilde{\alpha }}_{i}$ are linearly dependent on *B*/*J* ratio and ${\alpha }_{i}(B/J)={\tilde{\alpha }}_{i}(-B/J)$. Here, we can see that these two states are orthogonal and, thereby, the tunnelling between them is impossible $\langle {\Psi }_{B\uparrow }|{\hat{H}}_{B\downarrow }|{\Psi }_{B\downarrow }\rangle =0$. Consequently, one can conclude that the switching of the magnetization in even-numbered chains should proceed much faster than in odd-numbered counterparts.

### Data Availability

The datasets generated and analysed during the current study are available from the corresponding author on reasonable request.

## Electronic supplementary material

Supplementary Information 
