# Supplementary material for: Switching of spins and entanglement in surface-supported antiferromagnetic chains
Source: Sci Rep. 2017 Jun 5;7:2759. doi: 10.1038/s41598-017-02972-x (PMC5459826; doi:10.1038/s41598-017-02972-x)
Supplement: Supplementary file 1 — Supplementary Information [file 41598_2017_2972_MOESM1_ESM.pdf]

Switching of spins and entanglement in surface-supported antiferromagnetic chains: supplementary information

Ilia N. Sivkov[1,2], Dmitry I. Bazhanov[1,3,4], and Valeri S. Stepanyuk[1]

[1]*Max Planck Institute of Microstructure Physics, Halle, 06120, Germany*

[2]*ETH Zürich / CSCS Lugano, Switzerland*

[3]*Faculty of Physics, Moscow State University, GSP-1, 119991 Moscow, Russia*

[4]*Institution of Russian Academy of Sciences, Dorodnicyn Computing Centre of RAS, Vavilov st. 40, 119333 Moscow, Russia*

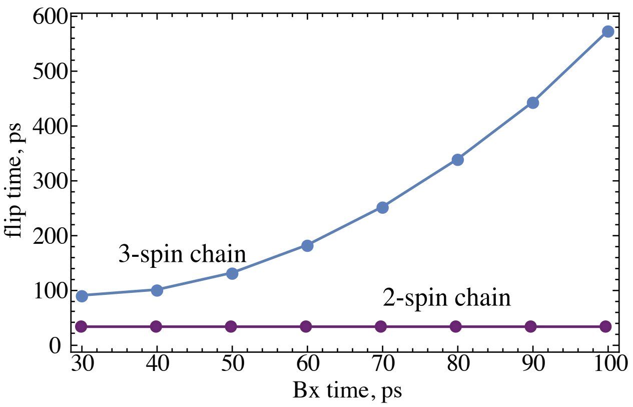

Figure S1: Dependence of a flip time of the 2 and 3-atomic chains on B_x pulse time t_0 (centre of a Gaussian function, describing the B_x pulse). The time of switch of B_z field is fixed and equals $t'_0 = 30\ ps$. It is seen that even-numbered chain is not influenced by the B_x pulse and flips earlier than the latter starts. At the same time the odd-numbered chain requires the pulse to start the switching process because the pulse breaks the symmetry with respect to z-axis and partially mixes 'up' and 'down' states of the chain. After that the relaxation process can start due to a damping term. Nevertheless, it is seen from the picture, that the main trend (even-numbered chains switch faster than the odd-numbered chains) is kept.

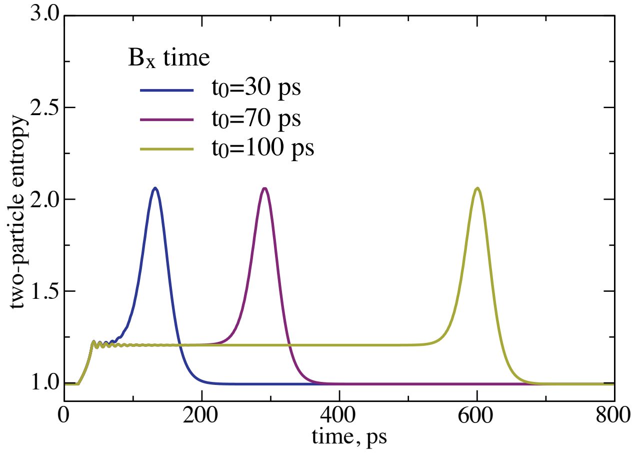

Figure S2: Two-particle Von Neumann entropy calculated between edge spins in the 5-atomic chain at different B_x times $t_0 = 30\ ps,\ 70\ ps,\ 100\ ps$, B_z time is $t_0' = 30\ ps$. The chain has significant entropy in the ground state before and after the switching. As in Fig. 1 the flip-time depends on the B_x pulse time.

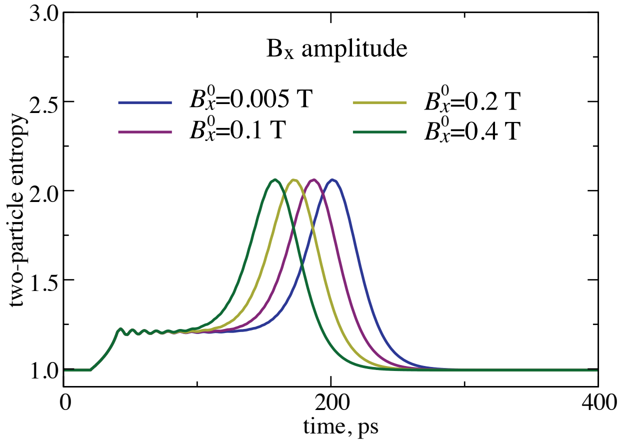

Figure S3: Two-particle Von Neumann entropy calculated between edge spins in the 5-atomic chain at different B_x^0 field amplitudes.

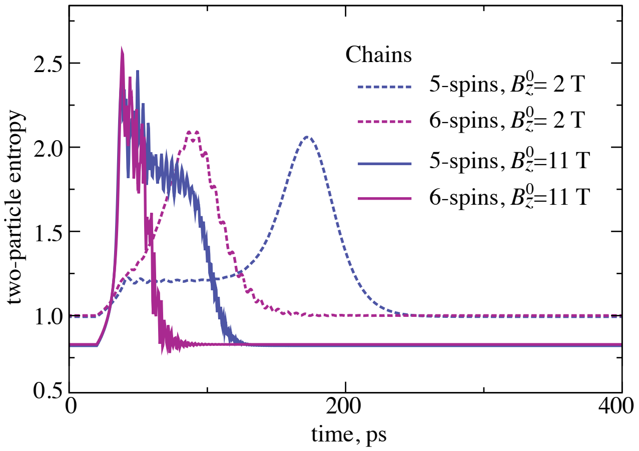

Figure S4: Two-particle Von Neumann entropy calculated between edge spins in the 5(blue) and 6(red)-atomic chains.
